# Supplementary material for: Conserved stromal–immune cell circuits secure B cell homeostasis and function
Source: Nat Immunol. 2023 May 18;24(7):1149–60. doi: 10.1038/s41590-023-01503-3 (PMC10307622; doi:10.1038/s41590-023-01503-3)
Supplement: Supplementary file 1 — Reporting Summary [file 41590_2023_1503_MOESM1_ESM.pdf]

nature portfolio

Corresponding author(s): Burkhard Ludewig, Natalia Pikor

Last updated by author(s): Mar 31, 2023

Reporting Summary

Nature Portfolio wishes to improve the reproducibility of the work that we publish. This form provides structure for consistency and transparency in reporting. For further information on Nature Portfolio policies, see our Editorial Policies and the Editorial Policy Checklist.

Statistics

For all statistical analyses, confirm that the following items are present in the figure legend, table legend, main text, or Methods section.

n/a	Confirmed	
☐	☒	The exact sample size (*n*) for each experimental group/condition, given as a discrete number and unit of measurement
☐	☒	A statement on whether measurements were taken from distinct samples or whether the same sample was measured repeatedly
☐	☒	The statistical test(s) used AND whether they are one- or two-sided<br>*Only common tests should be described solely by name; describe more complex techniques in the Methods section.*
☐	☒	A description of all covariates tested
☐	☒	A description of any assumptions or corrections, such as tests of normality and adjustment for multiple comparisons
☐	☒	A full description of the statistical parameters including central tendency (e.g. means) or other basic estimates (e.g. regression coefficient) AND variation (e.g. standard deviation) or associated estimates of uncertainty (e.g. confidence intervals)
☐	☒	For null hypothesis testing, the test statistic (e.g. *F*, *t*, *r*) with confidence intervals, effect sizes, degrees of freedom and *P* value noted<br>*Give P values as exact values whenever suitable.*
☒	☐	For Bayesian analysis, information on the choice of priors and Markov chain Monte Carlo settings
☒	☐	For hierarchical and complex designs, identification of the appropriate level for tests and full reporting of outcomes
☐	☒	Estimates of effect sizes (e.g. Cohen's *d*, Pearson's *r*), indicating how they were calculated

Our web collection on statistics for biologists contains articles on many of the points above.

Software and code

Policy information about availability of computer code

Data collection	FACSDiva (BD Biosciences, v8.0.1 and v9.0.1), FACSChorus (BD Biosciences, v1.3), ZEN blue (Zeiss, v3.3)
Data analysis	FlowJo (Treestar Inc., v10), R (v.4.0.0), CellRanger (v3.0.2), scater R/Bioconductor package (v.1.16.0), Seurat R package (v.4.0.1), clusterProfiler R/Bioconductor package (v.3.15.3), variancePartition R package (v.1.22.0), muscat R/Bioconductor package (v.1.6.0), CellPhone-DB (v.2.1.7), python v.3.7.0, Imaris (v9), Space Ranger software (v.1.3.1), Loupe Browser (v.6.2.0), SpatialExperiment R/Bioconductor package (v.1.6.1), spacexr R package (v.2.0.1). Code used for data analysis in this project is available at github (https://github.com/mluetge/CrossSLO_BRC_CXCL13)

For manuscripts utilizing custom algorithms or software that are central to the research but not yet described in published literature, software must be made available to editors and reviewers. We strongly encourage code deposition in a community repository (e.g. GitHub). See the Nature Portfolio guidelines for submitting code & software for further information.

Data

Policy information about availability of data

All manuscripts must include a data availability statement. This statement should provide the following information, where applicable:

- Accession codes, unique identifiers, or web links for publicly available datasets
- A description of any restrictions on data availability
- For clinical datasets or third party data, please ensure that the statement adheres to our policy

Ensembl GRCm38.9 and GRCh38.9 were used as reference genomes to build the indexes. The scRNA-seq and spatial transcriptomics data generated in this study have been deposited in the BioStudies database (www.ebi.ac.uk/biostudies/). Mouse scRNAseq data is available under accession code E-MTAB-11738, human lymph node data is available under E-MTAB-11710 and data from human palatine tonsils is available under E-MTAB-11715. Spatial transcriptomics data is available

under accession code E-MTAB-12468. Processed data files can be downloaded from the figshare platform (https://figshare.com) under 10.6084/m9.figshare.21221291 and explored via an interactive browser at https://immbiosg.github.io/FRCdataExplorer/.

Field-specific reporting

Please select the one below that is the best fit for your research. If you are not sure, read the appropriate sections before making your selection.

☒ Life sciences ☐ Behavioural & social sciences ☐ Ecological, evolutionary & environmental sciences

For a reference copy of the document with all sections, see nature.com/documents/nr-reporting-summary-flat.pdf

Life sciences study design

All studies must disclose on these points even when the disclosure is negative.

Sample size	No sample-size calculation was performed. Sample sizes were determined to be adequate based on the reproducibility between independent experiments and adequate cell numbers of each subset in the scRNA-seq data to run comparative analyses. Sample sizes for both single cell and experimental studies were based on our experience and common practise in the field (Nat Immunol. 2020 Jun;21(6):649-659).
Data exclusions	No data points were excluded.
Replication	For analysis of the performed scRNA-seq experiments no batch correction needed to be applied for any of the samples. All attempts at replication were successful. For all experimental analyses, all attempts at replication were successful.
Randomization	Randomization and control of covariants was not relevant in the setting of this exploratory study.
Blinding	Blinding was not performed since data analysis was explorative.

Reporting for specific materials, systems and methods

We require information from authors about some types of materials, experimental systems and methods used in many studies. Here, indicate whether each material, system or method listed is relevant to your study. If you are not sure if a list item applies to your research, read the appropriate section before selecting a response.

Materials & experimental systems

n/a	Involved in the study
☐	☒ Antibodies
☐	☒ Eukaryotic cell lines
☒	☐ Palaeontology and archaeology
☐	☒ Animals and other organisms
☐	☒ Human research participants
☒	☐ Clinical data
☒	☐ Dual use research of concern

Methods

n/a	Involved in the study
☒	☐ ChIP-seq
☐	☒ Flow cytometry
☒	☐ MRI-based neuroimaging

Antibodies

Antibodies used

Histology antibodies:
Anti-GFP polyclonal chicken (Aves Labs Inc., Cat#: GFP-1020, Lot#: GFP879484, 1:1000)
Anti-DsRed polyclonal rabbit (Takara Bio Clontech, Cat#: 632496, Lot#: 1805060, 1:1000)
Anti-human/mouse B220 eFluor450 (Thermo Scientific, Cat#: 48-0452-82, clone: RA3-6B2, Lot#: 2195593, 1:200)
Anti-mouse Lyve1 eFluor660 (Thermo Scientific, Cat#: 50-0443-82, clone:ALY-7, Lot#: 2205461, 1:200)
Anti-mouse CD4 AlexaFluor488 (BioLegend, Cat#: 100529, clone: RM4-5, Lot#: B243360 ,1:200)
Anti-mouse F4/80 AlexaFluor647 (BioLegend, Cat#: 123122, clone: BM8, Lot#: B265213, 1:200)
Anti-mouse PDPN syrian hamster (BioLegend, Cat#: 127402, clone: 8.1.1, Lot#: B228668, 1:300)
Anti-mouse CD21/35 APC (BioLegend, Cat#: 123412, clone: 7E9, Lot#: B256895, 1:500)
Anti-mouse MAdCAM1 Biotin (BioLegend, Cat#: 120706, clone: MECA-367, Lot#: B187519, 1:200)
Anti-mouse CD157 APC (BioLegend, Cat#: 140208, clone: BP-3, Lot#: B213863, 1:100)
Anti-mouse CD31 AlexaFluor647 (BioLegend, Cat#: 102516, clone: MEC13.3, Lot#: B308659, 1:200)
Anti-mouse PNAd Biotin (BioLegend, Cat#: 120804, clone: MECA-79, Lot#: B177144, 1:200)
Anti-mouse CD326/EPCAM AlexaFluor488 (BioLegend, Cat#: 118210, clone: G8.8, Lot#: B285223, 1:200)
Anti-mouse CXCL13 Biotin polyclonal goat (R&D Systems ,Cat#: BAF470, Lot#: DAD0314121, 1:100)
Anti-mouse CCL21 Biotin polyclonal goat (R&D Systems, Cat#: BAF457, Lot#: BEO0819071, 1:100)
Anti-mouse TRANCE/RANKL polyclonal goat (R&D Systems, Cat#: AF462, Lot#: CKN0320092, 1:200)
Anti-human / mouse SMA Cy3 (Sigma, Cat#: C6198, clone:1A4, Lot#: 0000116745, 1:400)
Anti-human CD3 polyclonal rabbit (Dako, Cat#: A045201-2, Lot#: 20061852, 1:200)
Anti-human CD20 AlexaFluor488 (Thermo Scientific, Cat#: 53-0202-82, clone: L26, Lot#: 2210882, 1:200)

Anti-human PDPN (Thermo Scientific, Cat#: 14-9381-82, clone: NZ-1.3, Lot#: 2400405, 1:200)
Anti-human Clusterin (BD Biosciences, Cat#: 552886, clone: E5, Lot#: 9346561, 1:200)
Anti-human CXCL13 (R&D Systems, Cat#: MAB801, clone: 53610, Lot#: BJT0819081, 1:100)
Anti-human CCL19 polyclonal rabbit (Abcam, Cat#: ab221704, Lot#: GR3409392-1, 1:100)
Anti-Chicken AlexaFluor488 polyclonal (Jackson ImmunoResearch, Cat#: 703-545-155, Lot#: 151901, 1:1000)
Anti-Goat AlexaFluor488 polyclonal (Jackson ImmunoResearch, Cat#: 705-545-003, Lot#: 148783, 1:1000)
Anti-Goat AlexaFluor647 polyclonal (Jackson ImmunoResearch, Cat#: 705-605-003, Lot#: 153846, 1:1000)
Anti-Mouse Cy3 polyclonal (Jackson ImmunoResearch, Cat#: 715-165-150, Lot#: 155993, 1:1000)
Anti-Rabbit AlexaFluor488 (Jackson ImmunoResearch, Cat#: 711-545-152, Lot#: 158217, 1:1000)
Anti-Rabbit Cy3 polyclonal (Jackson ImmunoResearch, Cat#: 711-165-152, Lot#: 157936, 1:1000)
Anti-Syrian hamster AlexaFluor488 (Jackson ImmunoResearch, Cat#: 107-545-142, Lot#:150847, 1:100)
Anti-Rat AlexaFluor647 (Jackson ImmunoResearch, Cat#: 712-606-150, Lot#: 150018, 1:1000)
Streptavidin AlexaFluor488 (Jackson ImmunoResearch, Cat#: 016-540-084, Lot#: 138230, 1:1000)
Streptavidin Cy3 (Jackson ImmunoResearch, Cat#: 016-160-084, Lot#: 141873, 1:1000)
Streptavidin-AlexaFluor647 (Jackson ImmunoResearch, Cat#: 016-600-984, Lot#: 141873, 1:1000)

Flow cytometry and cell sorting antibodies:
Anti-mouse CD157 BV786 (BD Biosciences, Cat#: 741012, clone: BP-3, Lot#: 1047515, 1:200)
Anti-mouse CD45.2 BV605 (BioLegend, Cat#: 109841, clone: 104, Lot#: B310111, 1:200)
Anti-mouse CD31 AlexaFluor647 (BioLegend, Cat#: 102516, clone: MEC13.3, Lot#: B308659, 1:200)
Anti-mouse PDPN PE-Cy7 (BioLegend, Cat#: 127412, clone: 36899, Lot#: B310444, 1:200)
Anti-mouse CD21/35 Pacific Blue (BioLegend, Cat#: 123414, clone: 7E9, Lot#: B294084, 1:200)
Anti-mouse MAdCAM1 Biotin (BioLegend, Cat#: 120706, clone: MECA-367, Lot#: B187519, 1:200)
Anti-mouse SCA1 APC-Cy7 (BioLegend, Cat#: 108126, clone: D7, Lot#: B253002, 1:200)
Anti-mouse CD19 APC-Cy7 (BioLegend, Cat#: 115530, clone: 6D5, Lot#: B290859, 1:200)
Anti-mouse CD38 PE (BioLegend, Cat#: 102708, clone: 90, Lot#: 2209694, 1:200)
Anti-mouse/human GL7 AlexaFluor488 (BioLegend, Cat#: 144612, clone: GL-7, Lot#: B256896, 1:200)
Anti-mouse CD4 PE (BioLegend, Cat#: 116006, clone: RM4-5, Lot#: B186627, 1:200)
Anti-mouse CD11b AlexaFluor647 (BioLegend, Cat#: 101218, clone: M1/70, Lot#: B236178, 1:200)
Anti-human CD45 FITC (BD Biosciences, Cat#: 555482, clone: H130, Lot#: 8120939, 1:200)
Anti-human PDPN PE (Thermo Scientific, Cat#: 12-9381-42, clone: NZ-1.3, Lot#: 4332768, 1:200)
Anti-human CD31 APC (Thermo Scientific, Cat#: 17-0319-42, clone: WM59, Lot#: 1976592, 1:200)
Anti-human EPCAM FITC (BioLegend, Cat#: 324203, clone: 9C4, Lot#: B261791, 1:200)
Anti-human CD14 PE-Cy7 (BioLegend, Cat#: 301813, clone: MSE2, Lot#: B231081, 1:200)
Anti-human CD3 FITC (BioLegend, Cat#: 300440, clone: UCHT1, Lot#: B279209, 1:200)
Anti-human CD19 APC/Fire 750 (BioLegend, Cat#: 302258, clone: HIB19, Lot#: B242981, 1:200)
Anti-human CD45 PeCy7 (Thermo Scientific, Cat#:25-0459-42, Clone: HI30, Lot#: 2079970, 1:200)
Anti-human CD31 Biotin (Thermo Scientific, Cat#:13-0319-82, Clone: WM59, Lot#: 1994108, 1:200)
Anti-human CD235a PeCy7 (BioLegend, Cat#:349112, Clone: HI264, Lot#: B274230, 1:200)
Anti-human CD34 FITC (BioLegend, Cat#:343504, Clone: 581, Lot#: B356958, 1:200)
Streptavidin-BV711 (BioLegend, Cat#: 405241, Lot#: B332218, 1:1000)

Validation

All antibodies came from commercial vendors, and were validated by the manufacturers on their official website. For stainings that used a combination of primary and secondary antibodies, each primary antibody was additionally validated by performing control stains using the secondary antibody alone to ensure a specific signal.

Eukaryotic cell lines

Policy information about cell lines

Cell line source(s)	Vero ATCC CCL-18
Authentication	The functionality of the cell line was confirmed in our labtoratory, as demonstrated by the ability to be infected by VSV.
Mycoplasma contamination	Vero cell lines tested negative for mycoplama contamination in our lab.
Commonly misidentified lines (See ICLAC register)	No misidentified lines were used

Animals and other organisms

Policy information about studies involving animals; ARRIVE guidelines recommended for reporting animal research

Laboratory animals	Experiments were performed with 6 to 10 week-old mice (males and females). All animals were housed in individually ventilated cages under conventional specific pathogen-free conditions, maintaining a 12 hour light/dark cycle, 22ºC ambient temperature and 45/50% humidity. All mouse strains were on a C57BL/6N Charles River genetic background. The C57BL/6N-Tg(Cxcl13-Cre/TdTomato) x R26-EYFP strain was described previously. C57BL/6N-Tg(Cxcl13-Cre)723Biat x B6.129-Il6tm1Jho (Cxcl13-Cre Il6fl/fl) mice were generated by crossing Cxcl13-Cre mice with B6.129-Il6tm1Jho (Il6fl/fl) mice that were described before.
Wild animals	none
Field-collected samples	none

Ethics oversight	Experiments were performed with 6- to 10-week-old mice (males and females) and were in accordance with Swiss federal and cantonal guidelines (Tierschutzgesetz) under permissions SG/26/2020 granted by the Veterinary Office of the Canton of St. Gallen.

Note that full information on the approval of the study protocol must also be provided in the manuscript.

Human research participants

Policy information about studies involving human research participants

Population characteristics	Detailed information is listed in Extended data Table 1 "Patient characteristics"
Recruitment	Tonsil samples were collected from adult patients suffering from obstructive sleep apnea (OSA) due to hyperplastic tonsils that underwent routine tonsillectomy at the Kantonsspital St. Gallen. Decision to perform surgery was made after clinical assessment by attending ENT physicians. Patient material was used following provision of informed consent.<br>Lymph node samples were collected from adult patients with benign LN swelling conditions that underwent routine lateral parotidectomy or transcervical excision of a cervical cyst at the Kantonsspital St. Gallen. Decision to perform surgery was made after clinical assessment by attending ENT physicians. Patient material was used following provision of informed consent.
Ethics oversight	Ethikkomission Ostschweiz (EKOS), Kantonsspital, Haus 37, 9007 St. Gallen

Note that full information on the approval of the study protocol must also be provided in the manuscript.

Flow Cytometry

Plots

Confirm that:

☒ The axis labels state the marker and fluorochrome used (e.g. CD4-FITC).

☒ The axis scales are clearly visible. Include numbers along axes only for bottom left plot of group (a 'group' is an analysis of identical markers).

☒ All plots are contour plots with outliers or pseudocolor plots.

☒ A numerical value for number of cells or percentage (with statistics) is provided.

Methodology

Sample preparation	A description of the sample preparation for flow cytometry and FACS sorting is detailed in the methods section.
Instrument	LSR Fortessa BD Biosciences, FACS Melody BD Biosciences
Software	FACSDiva and FACSChorus (both from BD) were used for data acquisition. FlowJO v10 (Treestar inc.) was used to analyze the data.
Cell population abundance	A test sample was prepared for testing the purity of sorted cells. The purity of the post-sort fraction was determined by flow cytometry using the LSR Fortessa 2.
Gating strategy	For all flow cytometric analyses, cells were first gated on FSC/SSC to exclude cell debris following by FSC-A/FSC-H to exclude doublets. Dead cells were excluded from analysis by gating on cells staining negative for a viability dye. A detailed gating strategy for stromal and immune cell populations is provided in the Extended data.

☒ Tick this box to confirm that a figure exemplifying the gating strategy is provided in the Supplementary Information.
